# Phosphorus-Containing Polymers as Sensitive Biocompatible Probes for ^31^P Magnetic Resonance

**DOI:** 10.3390/molecules28052334

**Published:** 2023-03-02

**Authors:** Lucie Kracíková, Ladislav Androvič, Iveta Potočková, Natalia Ziółkowska, Martin Vít, David Červený, Daniel Jirák, Richard Laga

**Affiliations:** 1 Institute of Macromolecular Chemistry, Czech Academy of Sciences, Heyrovského nám. 2, 162 06 Prague, Czech Republic; 2 Department of Polymers, Faculty of Chemical Technology, University of Chemistry and Technology, Technicka 5, 166 28 Prague, Czech Republic; 3 Institute for Clinical and Experimental Medicine, Vídeňská 1958/9, 140 21 Prague, Czech Republic; 4 Institute of Biophysics and Informatics, First Faculty of Medicine, Charles University, 121 08 Prague, Czech Republic; 5 Faculty of Health Studies, Technical University of Liberec, Studentská 1402/2, 461 17 Liberec, Czech Republic

**Keywords:** phosphorus-containing polymers, controlled polymerization, polymer probes, ^31^P magnetic resonance imaging

## Abstract

The visualization of organs and tissues using ^31^P magnetic resonance (MR) imaging represents an immense challenge. This is largely due to the lack of sensitive biocompatible probes required to deliver a high-intensity MR signal that can be distinguished from the natural biological background. Synthetic water-soluble phosphorus-containing polymers appear to be suitable materials for this purpose due to their adjustable chain architecture, low toxicity, and favorable pharmacokinetics. In this work, we carried out a controlled synthesis, and compared the MR properties, of several probes consisting of highly hydrophilic phosphopolymers differing in composition, structure, and molecular weight. Based on our phantom experiments, all probes with a molecular weight of ~3–400 kg·mol^−1^, including linear polymers based on poly(2-methacryloyloxyethyl phosphorylcholine) (PMPC), poly(ethyl ethylenephosphate) (PEEP), and poly[bis(2-(2-(2-methoxyethoxy)ethoxy)ethoxy)]phosphazene (PMEEEP) as well as star-shaped copolymers composed of PMPC arms grafted onto poly(amidoamine) dendrimer (PAMAM-*g*-PMPC) or cyclotriphosphazene-derived cores (CTP-*g*-PMPC), were readily detected using a 4.7 T MR scanner. The highest signal-to-noise ratio was achieved by the linear polymers PMPC (210) and PMEEEP (62) followed by the star polymers CTP-*g*-PMPC (56) and PAMAM-g-PMPC (44). The ^31^P *T*_1_ and *T*_2_ relaxation times for these phosphopolymers were also favorable, ranging between 1078 and 2368 and 30 and 171 ms, respectively. We contend that select phosphopolymers are suitable for use as sensitive ^31^P MR probes for biomedical applications.

## 1. Introduction

Phosphorus magnetic resonance (^31^P MR) including spectroscopy (^31^P MRS), imaging (^31^P MRI) and magnetic resonance spectroscopic imaging (^31^P MRSI) is a unique diagnostic technique used in human and experimental medicine, enabling non-invasive in vivo assessment of fundamental physiological processes at a cellular level without the use of ionizing radiation [1,2,3]. This is possible due to the presence of vital organophosphorous compounds in living organisms containing a stable phosphorus monoisotope (^31^P). The nuclei of ^31^P have favorable magnetic properties (*s* = ½, *γ* = 17.2 MHz∙T^−1^) that can be easily detected by MR scanners. In medical practice, ^31^P MR techniques are mostly used to reveal changes in the biochemistry of high-energy nucleotide coenzymes and phospholipid building blocks. Providing valuable insights into mitochondrial metabolism, membrane composition, and intracellular pH levels [3,4], this information can be utilized to investigate a wide range of severe diseases, including diabetes, stroke, heart failure, and cancer [5,6,7,8]. However, in contrast to conventional in vivo imaging of water and fat protons (^1^H), ^31^P MR measurements are hampered by the low physiological levels of organophosphorous compounds (~1000-fold lower abundance than compounds containing ^1^H) and the low MR sensitivity of ^31^P nuclei (~2.5-fold lower magnetic momentum compared to ^1^H). Therefore, ^31^P MRexperiments often require disproportionately long acquisition times in order to achieve adequate signal-to-noise ratios (SNRs) [1]. On the other hand, the range of chemical shifts at which most of the naturally occurring phosphorus compounds resonate is relatively wide—from -15 to 15 ppm (compared to the narrow 5 ppm window for ^1^H spectra)—thus enabling simple signal separation [9].

Other advantages of ^31^P MR include that it can also measure the biodistribution of systemically administered phosphorus-containing compounds (so-called probes). The acquired data can provide, for example, information about the accumulation of the probe in a specific organ or tissue, as well as the kinetics of its excretion from the body. However, from an application point of view, it is essential that the probe be non-toxic and non-immunogenic and provides a highly intense ^31^P MR signal that is distinguishable from the natural biological background. In addition, it is a great advantage if the probe is of a macromolecular or colloidal nature with a long biological half-life, but is eliminated from the body after performing its function. [10,11] The development of probes that meet these exacting criteria is therefore a major challenge in biomedical diagnostic research.

In recent decades, several different types of biocompatible phosphopolymers and phosphate-based colloids have been developed for various biomedical applications that meet the above criteria for use as potential exogenous ^31^P MR probes. However, systematic studies investigating the effect of their structure and composition on MR properties are lacking. Among the most exemplary materials are hydrophilic poly(organophosphazenes) (PPPs). These fully synthetic polymers, which feature a repeating phosphazene linkage (–N=P(R_1_R_2_)–) in the backbone, have been applied in many biomedical fields, including drug delivery, vaccination, and regenerative engineering [12,13]. They also offer excellent biocompatibility, biodegradability, and wide structural variability. Importantly, ^31^P nuclear magnetic resonance (NMR) studies have shown that PPP chemical shifts can be tuned by changing the types of R_1_ and R_2_ substituents (aliphatic vs. aromatic) and linkages (P–O vs. P–N vs. P–S bond) on the phosphorus atom in the polymer backbone, ensuring that the probe is spectroscopically distinguishable from biomolecules with phosphoester bonds [14,15]. Poly(phosphoesters) (PPEs) are also suitable synthetic phosphopolymers for ^31^P MR imaging. PPEs represent a broad class of biocompatible polymers characterized by the presence of repeating hydrolytically and enzymatically degradable phosphoester groups (–PO(OR_1_)–O–R_2_–O–) in the main polymer chain, thus resembling biomacromolecules such as nucleic acids or nucleotide-based coenzymes. Depending on the nature of the R_1_ side group, PPEs can have different chemical and physical properties, making them suitable in a wide range of biomedical applications [16,17]. Of the phosphorus-containing polymers, poly(2-methacryloyloxyethyl phosphorylcholine) (PMPC), a hydrophilic methacrylate-based polymer with a zwitterionic phosphorylcholine moiety (−OPO_3_^–^–CH_2_CH_2_–N^+^(CH_3_)_3_) on its side chain, has recently gained considerable attention due to its remarkable properties. PMPC is non-toxic, resistant to non-specific protein adsorption and cell adhesion, and has the ability to penetrate cell membranes [18]. In addition, 2-methacryloyloxyethyl phosphorylcholine (MPC) can be copolymerized with a wide range of functionalized monomers using a variety of polymerization techniques to tune the properties of the resulting polymer. MPC is widely utilized in drug delivery, antifouling coating technology, and biosensing [18,19,20,21]. It has been further demonstrated that replacing the phosphoester group (−OPO_3_––) in PMPC with a thiophosphoester group (−OPSO_2_––) leads to a significant chemical shift in its ^31^P MR spectrum while allowing the material to retain its advantageous chemical, biophysical, and biological properties [22]. Another notable material for ^31^P MR imaging is phytate (*myo*-inositol hexakisphosphate), a non-toxic biodegradable biomolecule that serves as a phosphorus reservoir (energy store) and source of *myo*-inositol (cell wall precursor) in plant seeds [23]. It has also been shown that calcium phytate nanoparticles doped with removable paramagnetic Fe^3+^ ions provide relatively high ^31^P MR signals, and that iron ions alter MR contrast in both ^1^H and ^31^P MR [24].

The aim of our study was to perform a controlled synthesis, and compare the MR properties, of several promising ^31^P MR probes composed of highly hydrophilic synthetic phosphopolymers. Synthesized polymer probes based on PPP, PPE, and PMPC were characterized using a 4.7 T MRI scanner and a 1.5 T MR relaxometer. The effects of composition, polymer chain architecture, and molecular weight on ^31^P MRS/MRI/MRSI signal intensity and *T*_1_ and *T*_2_ ^31^P relaxation times were evaluated. Our data demonstrate that, by tuning the physicochemical parameters of phosphopolymer chains, highly efficient probes can be prepared for future in vivo applications.

## 2. Results and Discussion

### 2.1. Synthesis and Physiochemical Characterization of Polymer Probes

Our requirements for determining a ^31^P MR probe of suitable composition were that the material would be water soluble, biocompatible, biodegradable, have a long biological half-life, and be structurally capable of carrying a large amount of phosphorus. Hydrophilic phosphopolymers are candidates that meet these criteria. In particular, we focused on synthetic phosphorus-containing polymers composed of poly(2-methacryloxyethyl phosphorylcholines) (PMPCs), poly(organophosphazenes) (PPPs), and poly(phosphoesters) (PPEs). The exceptional biologically amenable properties of these polymers have been successfully deployed in a number of biomedical applications [12,16,18]. Our comparison of individual polymers focused on differences in composition, polymer-chain architecture, and molecular weight. We prepared linear PMPC-, PPE-, and PPP-based polymers (M_n_ ranging from 4 to 22 kg·mol^−1^) as well as star-shaped PMPC-based polymers (M_n_ ranging from 59 to 399 kg·mol^−1^) (see Table 1). While the linear polymers were prepared by the modification of previously described procedures (see below), the star-shaped polymers were synthesized de novo for the purposes of this comparative study. To ensure well-defined polymers, controlled radical or ionic polymerization techniques were used. This resulted in materials with a very narrow molecular weight distribution (Ð ≤ 1.2) as well as a large quantity of terminal functional groups for the purpose of possible post-polymerization modification, e.g., targeting units, fluorescent dyes, and therapeutics. A detailed description of the synthetic procedures and physicochemical methods used for preparation and characterization the polymer probes is provided in the Supplementary Information.

**Figure 1:**
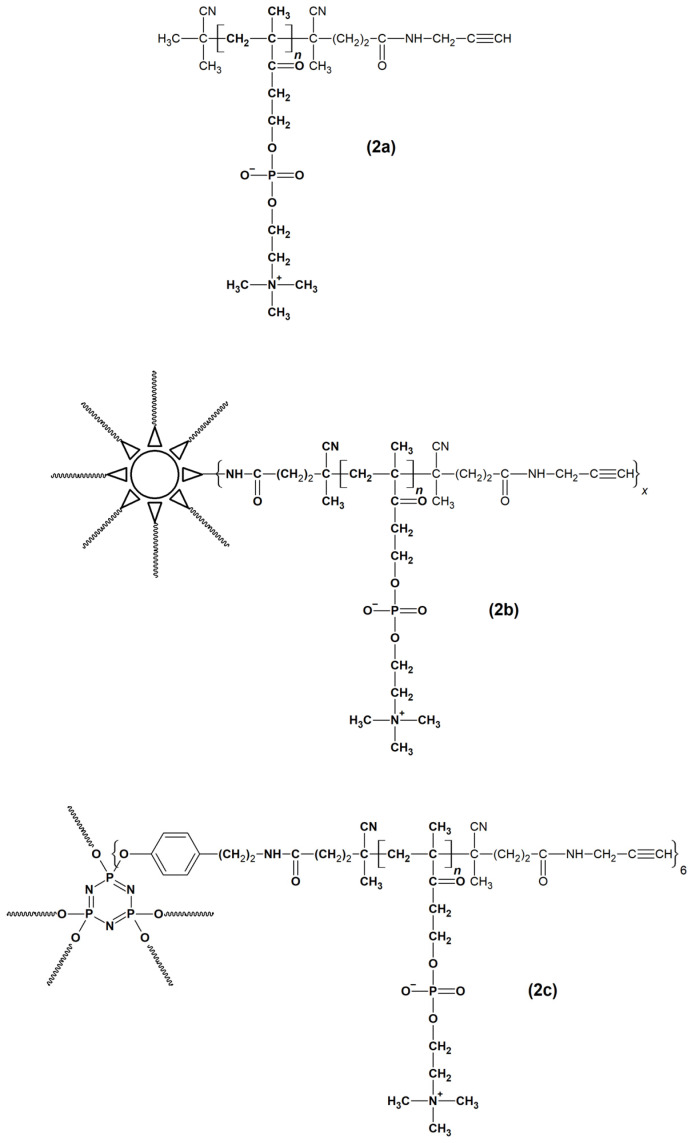
Chemical structures of ^31^P MR probes based on linear and star-shaped MPC-based polymers: (**2a**) IBN-PMPC-Pg, (**2b**) PAMAM-g-PMPC-Pg, and (**2c**) CTP-g-PMPC-Pg.

A linear PMPC polymer with a propargyl (Pg) end group was synthesized by reversible addition-fragmentation chain-transfer polymerization (RAFT) of a commercially available 2-methacryloxyethyl phosphorylcholine (MPC) monomer in the presence of a functionalized chain transfer agent (CTA-Pg) and an initiator (ACVA-Pg) at a [MPC]/[CTA-Pg]/[ACVA-Pg] ratio of 34:1:0.5. The dithiobenzoate (DTB) group on the other side of the polymer chain was either replaced by a non-reactive isobutyronitrile (IBN) group by a homolytic reaction with a high molar excess of AIBN to form the polymer IBN-PMPC-Pg (**2a**) or by an amine-reactive thiazolidine-2-thione (TT) group using a similar reaction with a functionalized azoinitiator (ACVA-TT) to yield the polymer precursor TT-PMPC-Pg. The molecular weight and dispersity of both polymers were ~19 kg·mol^−1^ and 1.06, respectively. The linear polymer precursor TT-PMPC-Pg was further attached via an aminolytic reaction to a 5^th^ generation poly(amidoamine) dendrimer (PAMAM); the molar ratio of the surface amino groups of the dendrimer to the terminal TT groups of the polymer was 2:1, which led to the binding of a sufficient amount of polymer arms and a relatively high conjugation efficiency. After removing the unreacted linear polymer on centrifugation membrane filters, a nearly monodisperse star polymer PAMAM-g-PMPC-Pg (**2b**) with ~20 polymer arms and a molecular weight of ~400 kg·mol^−1^ was obtained. A star polymer with a cyclotriphosphazene (CTP)-derived core was produced using the “grafting from” approach via the RAFT mechanism with a hexavalent chain transfer agent (CTP-(CTA-COOH)_6_). Polymerization of the MPC monomer in the presence of CTP-(CTA-COOH)_6_ led to the formation of a six-armed star polymer with terminal DTB groups, which were subsequently replaced by Pg groups by reaction with a functionalized azoinitiator (ACVA-Pg). The resulting star polymer CTP-g-PMPC-Pg (**2c**) was characterized by low dispersity (1.1) and a molecular weight of ~60 kg·mol^−1^. The chemical structures of the MPC-based linear and star polymers are shown in Figure 1, their SEC profiles are shown in Figure S5, and their characteristics are summarized in Table 1.

**Figure 2:**
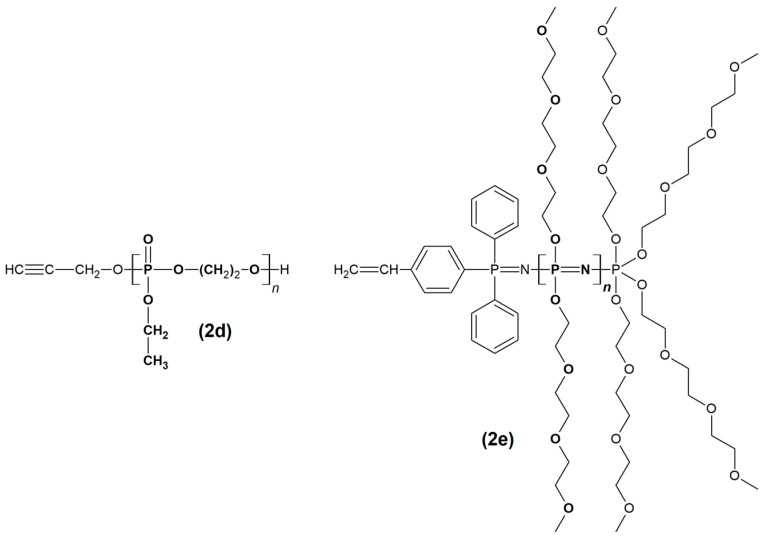
Chemical structures of ^31^P MR probes based on linear PPE and PPP polymers: (**2d**) PEEP-Pg and (**2e**) PMEEEP-Vi.

PAMAM-g-PMPC-Pg (2b) with ~20 polymer All MPC-based phosphopolymers were prepared from a commercial monomer (MPC) by controlled solution radical polymerization (RAFT). Linear PPE and PPP were obtained by anionic or cationic polymerization of monomers specifically synthesized for this purpose. Linear PPE was produced by DBU-initiated ring-opening anionic polymerization of 2-ethoxy-2-oxo-1,3,2-dioxaphospholane in the presence of 2-propyn-1-ol. Under strictly anhydrous conditions, water-soluble biodegradable poly(ethyl ethylene phosphate) (PEEP) with a terminal Pg group PEEP-Pg (**2d**) was obtained. The molecular weight and dispersity of the polymer were ~4 kg·mol^−1^ and 1.03, respectively. The preparation of hydrophilic PPP required not only the synthesis of the monomer, but also the functionalized initiator and subsequent post-polymerization modification of the generated reactive precursor. By the action of the functionalized cationic initiator 4-(dichlorodiphenylphosphino)styrene, the trichloro(trimethylsilyl)phosphoranimine monomer was converted to a reactive poly(dichlorophosphazene) precursor with a terminal vinyl group, which was further modified with 2-(2-(2-methoxyethoxy)ethoxy)ethanol in the presence of NaH to form the final water-soluble poly[bis(2-(2-(2-methoxyethoxy)ethoxy)]phosphazene (PMEEEP-Vi) (**2e**). By ensuring the high purity of all reactants and maintaining anhydrous conditions throughout the polymerization process, it was possible to produce a linear polymer with a molecular weight of 22 kg·mol^−1^ and a dispersity of 1.2. The chemical structures of the PPE- and PPP-based linear polymers are shown in Figure 2, their SEC profiles are shown in Figure S1, and their SEC characteristics are summarized in Table 1.

### 2.2. MR Properties of Polymer Probes

**Figure 3:**
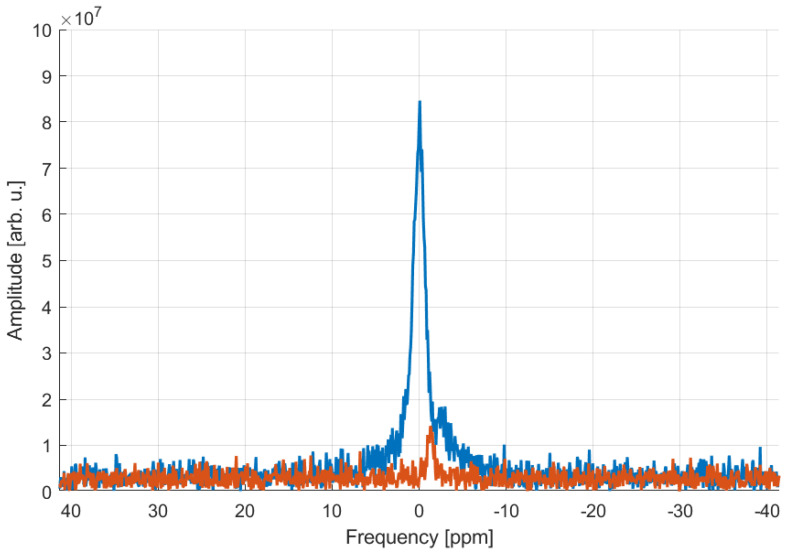
^31^P MR spectra of the PMPC probe and the reference PCr. The blue line represents the signal from two phantoms containing the PMPC and PCr, while the red line represents the signal from the reference PCr only.

High MR sensitivity (signal-to-noise ratio) is essential especially in lower magnetic fields close to those used in clinical medicine. MR spectroscopic methods, including MRSI-based sequences, are much more sensitive compared to conventional MR imaging sequences (e.g., RARE), which we verified on the PMPC probe at different repetition times (see Figures S7 and S8). Therefore, we used spectroscopic sequences based on chemical shift imaging (CSI) to image the phantoms. In addition, the CSI signal can be easily monitored in different regions using a spectroscopic signal matrix and is therefore relevant for future in vivo applications where probe localization will be required. Taking into consideration all of the MR parameters measured, IBN-PMPC-Pg (**2a**) and PMEEEP-Vi (**2e**) produced the best results. The SNR values calculated for both probes were of a sufficiently high sensitivity for ^31^P MR detection. Furthermore, phosphorus MR relaxation times were within the range applicable for in vivo use. The shorter *T*_1_ and longer *T*_2_ relaxation times for IBN-PMPC-Pg (**2a**) make it the most promising of the two probes. Its shorter *T*_1_ relaxation time allows for fast scanning and its longer *T*_2_ relaxation time enables to obtain a stronger MR signal. The results for PAMAM-*g*-PMPC-Pg (**2b**) and CTP-*g*-PMPC-Pg (**2c**) were similar. Both had long *T*_1_ and *T*_2_ relaxation times and relatively low SNR values, probably because the repetition time (TR) used was much shorter (TR = 500 ms) than optimal values. Considering measurements with a longer TR would increase the measurement time and the animal/patient burden related to it, a short TR was implemented for all probes to prioritise clinical applicability. For the same reason, the lowest SNR value was for PEEP-Pg (**2d**), with a *T*_1_ relaxation time approximately double the times of the first two polymer probes mentioned above. If the optimal TR (approx. 5 × *T*_1_) had been chosen, measurements would have been unacceptably long. Given that the chemical composition of probes (**2a–c**) and the structural motifs of probes (**2d** and **2e**) were similar to probes previously documented by our study group [20], we assumed their ^1^H relaxation times would also be comparable. Therefore, we did not expect an effect on MR contrast in anatomical (reference) ^1^H MRI. This assumption was confirmed when measuring reference ^1^H MR images for ^31^P CSI measurements, where we found no artifacts corresponding to shortened relaxation times. Although we could not find a correlation between the physicochemical parameters (composition, morphology, molecular weight) of the polymer probes and their MR properties, we can conclude that all of the prepared materials can potentially be used as exogenous probes for experimental and clinical phosphorus MR imaging, despite the fact that the presented macromolecules provide a ^31^P MR signal at approximately 0 ppm as for most phosphoester group-containing biomolecules. The presented polymer probes applied at higher concentrations provide a higher signal intensity than the natural biological background and therefore they should be detected even at the same chemical shift. To confirm these expectations, we performed a model experiment in which we measured MR spectra of a selected polymer probe (PMPC) and phosphocreatine (PCr), as a representative of one of the most abundant phosphorus-containing biomolecules. The molar concentration of phosphorus (*c*^P^) in the PMPC probe was set to 100 mmol·L^−1^, which we know is not toxic to mice [22], and the *c*^P^ in PCr was 27 mmol·L^−1^, which corresponds to the average content of PCr in human skeletal muscle [25]. After obtaining non-localized MR spectra of both compounds, the tube containing PMPC was removed from the instrument, after which further measurement of the reference PCr was performed. The measurement results clearly show that the ^31^P MR signal (SNR) of the polymer probe is more than 5-fold higher than that of the reference PCr (see Figure 3). These data suggest that the prepared polymer probes should be distinguishable from the biological background under in vivo conditions.

An alternative solution could be to substitute the phosphoesters in their structures with thiophosphoesters, which would lead to the production of an ^31^P MR signal with a different chemical shift (Δ*δ* ~ 56 ppm) than that provided by the biological background [22]. However, this approach would require the de novo synthesis of all monomers and their subsequent conversion to high-molecular-weight products, which was not the subject of this study.

## 3. Materials

### 3.1. Chemicals

3-Amino-1-propyne, 2-chloro-2-oxo-1,3,2-dioxophospholane, 1,8-diazabicyclo [5.4.0]undec-7-ene (DBU), *N*,*N*′-dicyclohexylcarbodiimide (DCC), diethylene glycol dimethyl ether (diglyme), 4-dimethylaminopyridine (DMAP), 1-ethyl-3-(3-dimethylaminopropyl)carbodiimide (EDC), hexachloroethane, hexamethyldisilazane, 2-methacryloyloxyethyl phosphorylcholine (MPC), 2-(2-(2-methoxyethoxy)ethoxy)ethanol, 2-propyn-1-ol, thiazolidine-2-thione (TT), and triethylamine were purchased from TCI Europe, Zwijndrecht, Belgium. 4,4′-Azobis(4-cyanopentanoic acid) (ACVA), 2,2′-Azobis(2-methylpropionitrile) (AIBN), *N*-(*tert*-butoxycarbonyl)tyramine, butyl lithium, cesium carbonate, 4-cyano-4-(phenylcarbonothioylthio)pentanoic acid (CTA-COOH), 4-(diphenylphosphino)styrene, poly(amidoamine) dendrimer, ethylenediamine core, G5 (pAMAM), hexachlorocyclotriphosphazene (CTP-(Cl)_6_), phosphorus trichloride, and sulfuryl chloride were purchased from Sigma-Aldrich, Prague, Czech Republic. Toluene was pre-dried over Na (in the presence of benzophenone) and dichloromethane over P_2_O_5_ and then distilled into a flask filled with molecular sieves (4 Å). All other solvents of HPLC grade (obtained from VWR International, Stříbrná Skalice, Czech Republic) were dried over a layer of activated molecular sieves (4 Å) before use.

### 3.2. Synthesis of Monomers, Initiators and Chain Transfer Agents

**Scheme 1:**
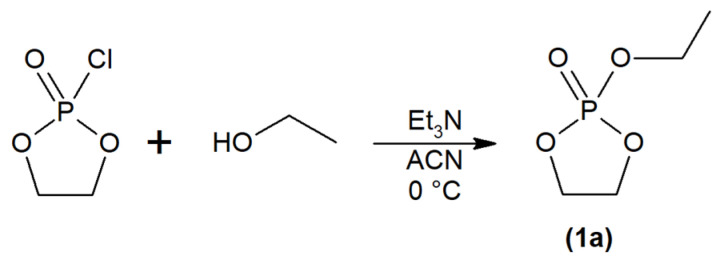
Reaction scheme for the synthesis of 2-ethoxy-2-oxo-1,3,2-dioxaphospholane (**1a**).

*(**1a**) 2-ethoxy-2-oxo-1,3,2-dioxaphospholane* monomer was synthesized by reacting 2-chloro-2-oxo-1,3,2-dioxophospholane with ethanol in acetonitrile in the presence of triethylamine [26] (see Scheme 1).

**Scheme 2:**
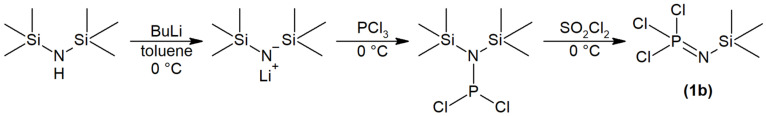
Reaction scheme for the synthesis of trichloro(trimethylsilyl)phosphoranimine (**1b**).

*(**1b**) Trichloro(trimethylsilyl)phosphoranimine* monomer was synthesized according to a modified procedure described elsewhere [27]. Briefly, a solution of butyl lithium (2.5 m, 5.0 mmol, 2.0 mL) in hexanes was added dropwise to hexamethyldisilazane (5.0 mmol, 1.05 mL) in dry toluene (20 mL) at 0 °C. The reaction mixture was then stirred for 1 h at r.t. To this solution, PCl_3_ (5.0 mmol, 0.44 mL) was added dropwise; the mixture was stirred at 0 °C for 30 min and then for another 1 h at r.t. Finally, SO_2_Cl_2_ (5.1 mmol, 0.41 mL) was added dropwise and the solution stirred at 0 °C for 1 h. The crude monomer in toluene solution was used directly in the polymerization reaction. For the reaction scheme, see Scheme 2.

**Scheme 3:**
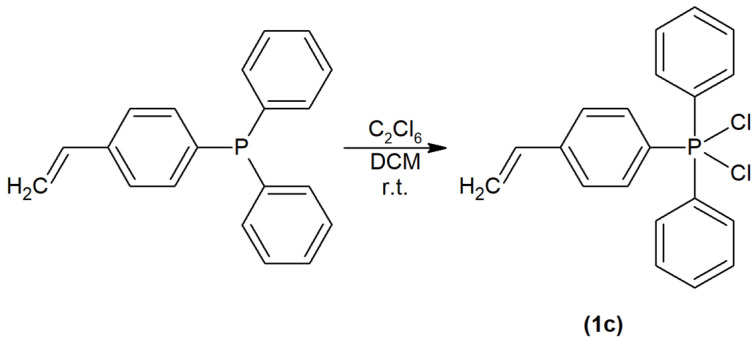
Reaction scheme for the synthesis of 4-(dichlorodiphenyphosphino)styrene (**1c**).

*(**1c**) 4-(Dichlorodiphenyphosphino)styrene* cationic initiator was generated by reacting 4-(diphenylphosphino)styrene (0.1 mmol, 29.0 mg) with hexachloroethane (0.11 mmol, 26.0 mg) in dry dichloromethane (1.5 mL) according to a previously described procedure [28] (see Scheme 3). The crude initiator in DCM solution was used in the polymerization reaction without further purification.

**Scheme 4:**
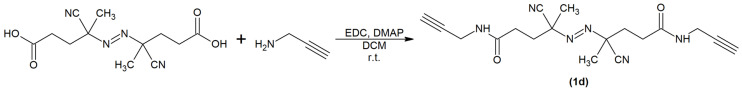
Reaction scheme for the synthesis of 4-cyano-4-(1-cyano-3-ethynylcarbamoyl-1-methylpropylazo)-N-ethynyl-4-methylbutyramide (**1d**).

**Scheme 5:**
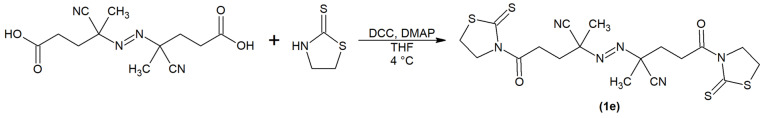
Reaction scheme for the synthesis of 2-[1-cyano-1-methyl-4-oxo-4-(2-thioxo-thiazolidin-3-yl)-butylazo]-2-methyl-5-oxo-5-(2-thioxothiazolidin-3-yl)-pentanenitrile (**1e**).

*(**1d**) 4-Cyano-4-(1-cyano-3-ethynylcarbamoyl-1-methylpropylazo)-N-ethynyl-4-methylbutyramide (ACVA-Pg)* radical initiator was synthesized by reacting ACVA with 3-amino-1-propyne in dichloromethane in the presence of EDC and DMAP [29] (see Scheme 4).

(**1e**) *2-[1-Cyano-1-methyl-4-oxo-4-(2-thioxo-thiazolidin-3-yl)-butylazo]-2-methyl-5-oxo-5-(2-thioxothiazolidin-3-yl)-pentanenitrile (ACVA-TT)* radical initiator was prepared by reacting ACVA with TT in tetrahydrofuran in the presence of DCC and DMAP [30] (see Scheme 5).

**Scheme 6:**
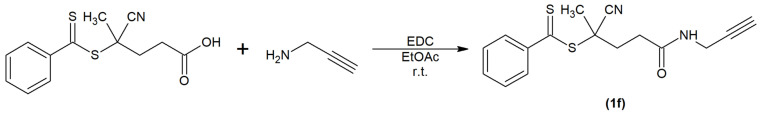
Reaction scheme for the synthesis of 2-cyano-5-oxo-5-[(prop-2-yn-1-yl)amino]pentan-2-yl benzenecarbodithioate (**1f**).

*(**1f**) 2-cyano-5-oxo-5-[(prop-2-yn-1-yl)amino]pentan-2-yl benzenecarbodithioate (CTA-Pg)* RAFT agent was prepared by reacting CTA-COOH with 3-amino-1-propyne in ethyl acetate in the presence of EDC [29] (see Scheme 6).

**Scheme 7:**
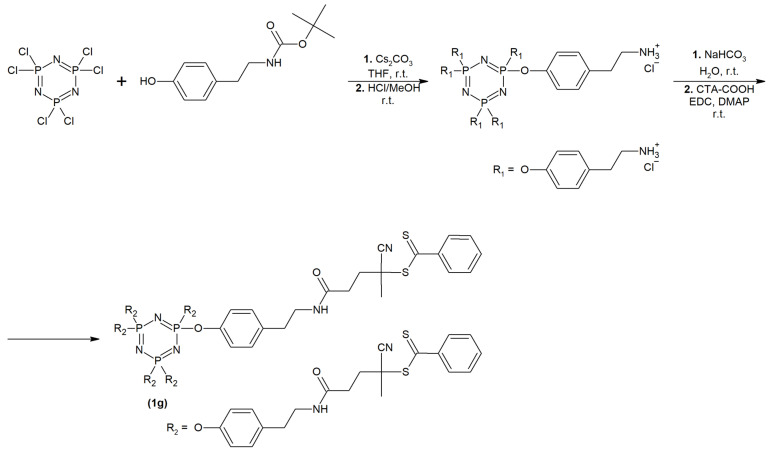
Reaction scheme for the synthesis of CTP-(DTB)6 (**1g**).

*(**1g**) CTP-(DTB)_6_* hexavalent RAFT reagent was synthesized using a multistep reaction starting with CTP-(Cl)_6_, which was converted to hexakis{4-[2-(tert-butoxycarbonyl)aminoethyl]phenoxy}-cyclotriphosphazene by condensation reaction with *N*-(*tert*-butoxycarbonyl)tyramine in the presence of Cs_2_CO_3_. This was followed by deprotection of its amino group in methanolic HCl and subsequent conjugation with CTA-COOH in the presence of EDC and DMAP [31]. For the reaction scheme, see Scheme 7.

### 3.3. Synthesis of Polymer Probes

**Scheme 8:**
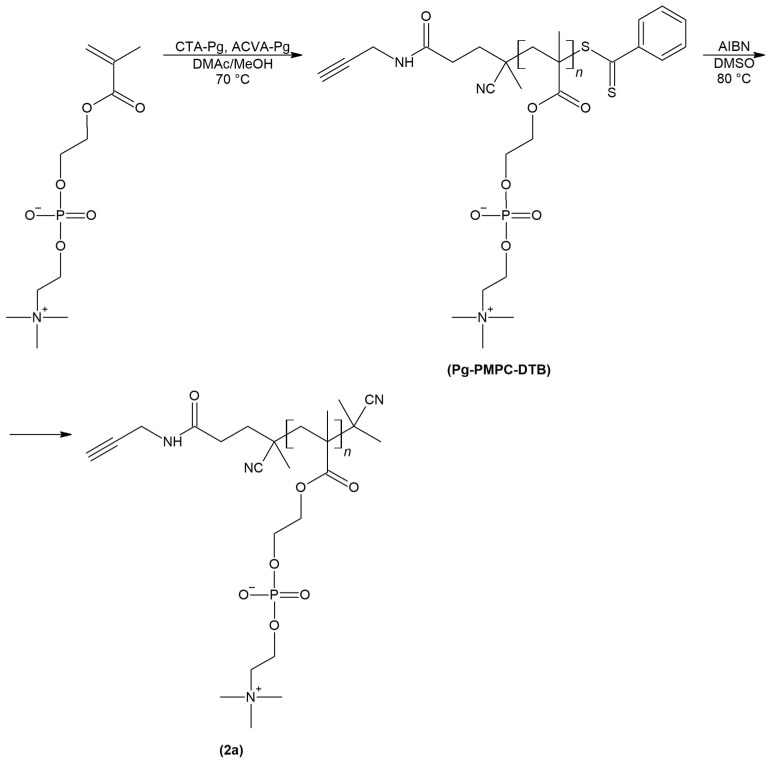
Reaction scheme for the synthesis of poly(2-methacryloyloxyethyl phosphorylcholine) linear polymer with a terminal propargyl group (**2a**).

*(**2a**) Poly(2-methacryloyloxyethyl phosphorylcholine) (PMPC)* linear polymer with a terminal propargyl (Pg) group was synthesized according to a procedure described in one of our previous papers [7]. Briefly, a mixture of CTA-Pg (41.4 mg, 105.0 µmol) and ACVA-Pg (18.6 mg, 52.5 µmol) was dissolved in 0.780 mL of dimethylacetamide (DMAc) and added to a solution of MPC (1.050 g, 3.57 mmol) in 2.775 mL of methanol. The solution was thoroughly bubbled with argon and polymerized in a sealed glass ampoule at 70 °C for 16 h. The polymerization mixture was then precipitated into 40 mL of acetone. The solid content was filtered off, dissolved in methanol, and re-precipitated into the same precipitant. After drying under vacuum, 440 mg (42 %) of PMPC-Pg polymer was obtained in the form of a pink powder. The number-average molecular weight (*M*_n_) and dispersity (*Ð*) were 18.5 kg·mol^−1^ and 1.06, respectively. The molar content of the dithiobenzoate (DTB) end groups was 41.3 µmol·g^−1^, which corresponds to an average number of 1.0 DTB groups per polymer chain. The DTB end groups were subsequently converted to isobutyronitrole (IBN) or thiazolidine-2-thione (TT) groups by reacting PMPC-Pg (216.2 mg, 11.7 µmol DTB groups) with AIBN (38.4 mg, 234.0 µmol) or ACVA-TT (112.8 mg, 234.0 µmol) at 80 °C for 3 h in DMSO (0.432 mL) in a sealed pressure tube. After cooling to r.t., the reaction mixtures were purified by column chromatography using a Sephadex LH-20 cartridge in a methanol-DMSO mixture. The purified polymers were isolated by precipitation into acetone (40 mL). The solid contents were centrifuged and dried under vacuum to give 148.0 mg of IBN-PMPC-Pg and 132.0 mg of TT-PMPC-Pg as a white and a yellow powder, respectively. The *M*_n_ and *Ð* of IBN-PMPC-Pg were 18.8 kg mol^−1^ and 1.06, respectively; the *M*_n_ and *Ð* of TT-PMPC-Pg were 19.0 kg·mol^−1^ and 1.06, respectively. The molar content of the TT end groups was 42.1 µmol·g^−1^, which corresponds to an average number of 0.8 TT groups per polymer chain. ^31^P NMR (162 MHz, D_2_O): *δ* 0.16 (s) ppm. The reaction scheme for the preparation of polymer (**2a**) is shown in Scheme 8.

**Scheme 9:**
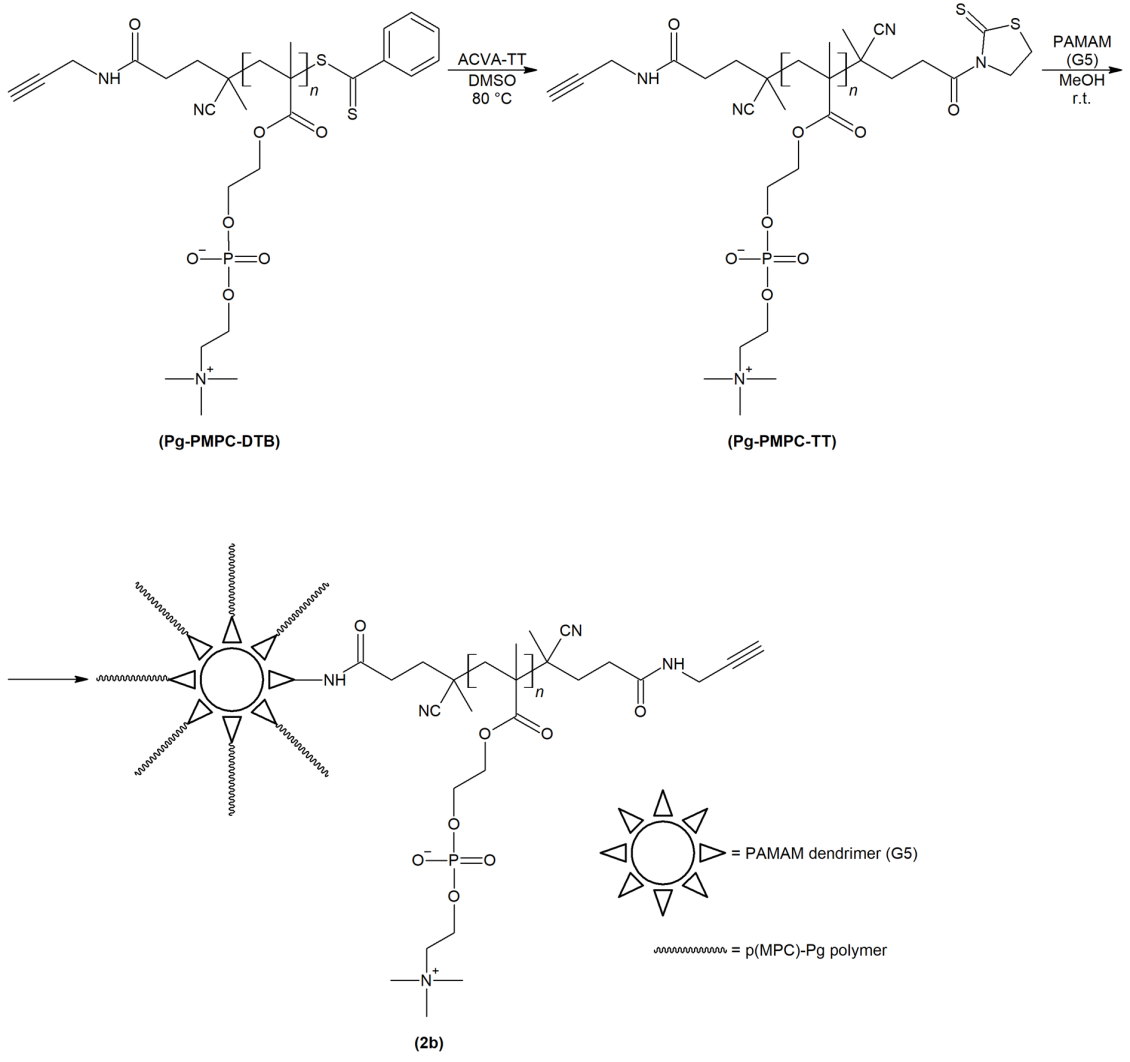
Reaction scheme for the synthesis of poly(amidoamine)-graft-poly(2-methacryloyloxyethyl phosphorylcholine) star copolymer with terminal Pg groups (**2b**).

*(**2b**) Poly(amidoamine)-graft-poly(2-methacryloyloxyethyl phosphorylcholine) (PAMAM-g-PMPC)* star copolymer, with terminal Pg groups and amide bonds between the PAMAM core and the PMPC arms, was prepared by reacting a two-fold molar excess of the PAMAM dendrimer (G5) with the TT-PMPC-Pg polymer as follows: TT-PMPC-Pg (83.1 mg, 3.5 µmol) was dissolved in dry methanol (0.830 mL) and mixed with 31.3 µL of stock solution (50 mg·mL^−1^) from PAMAM (1.6 mg, 7.0 µmol) in methanol. The solution was stirred overnight at r.t. The reaction mixture was precipitated into diethyl ether and the solid content then dissolved in PBS buffer (0.15 M, pH 7.4). The resulting star copolymer was separated from the unreacted linear polymer by membrane filtration using RC centrifugal filter units with MWCO 100 kDa in PBS (4×) and in H_2_O (2×). The purified star copolymer (PAMAM-*g*-PMPC-Pg) was isolated from the aqueous solution by lyophilization to give 50 mg of white powder. The *M*_n_ and *Ð* were 398.8 kg·mol^−1^ and 1.10, respectively. ^31^P NMR (162 MHz, D_2_O): *δ* 0.16 (s) ppm. The reaction scheme for the preparation of polymer (**2b**) is shown in Scheme 9.

**Scheme 10:**
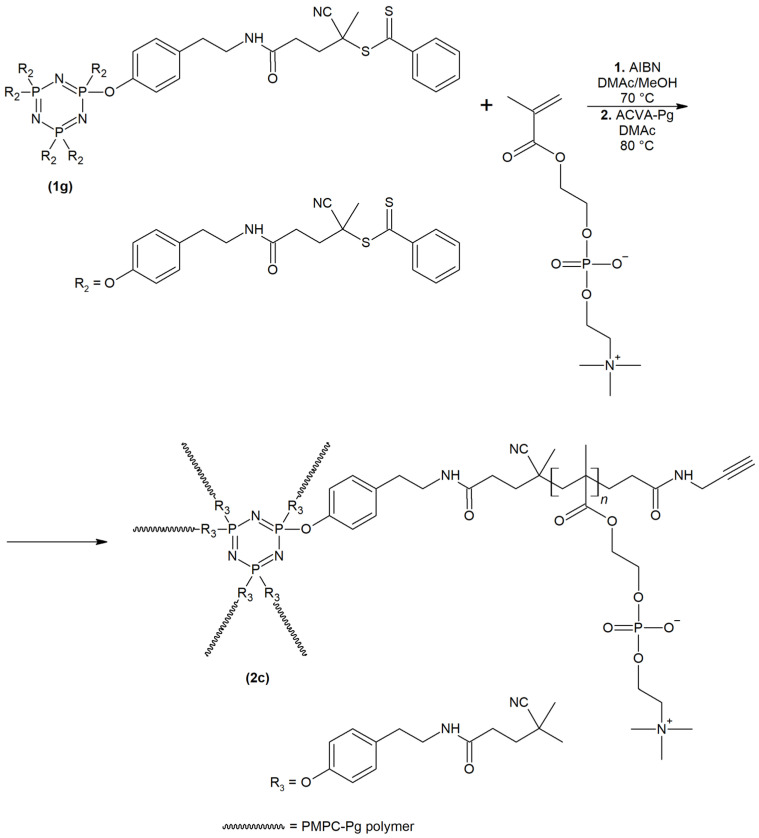
Reaction scheme for the synthesis of hexakis[4-(2-aminoethyl)phenoxy]cyclotriphosphazene-graft-poly(2-methacryloyloxyethyl phosphorylcholine) star copolymer with terminal Pg groups (**2c**).

*(**2c**) Hexakis [4-(2-aminoethyl)phenoxy]cyclotriphosphazene-graft-poly(2-methacryloyloxyethyl phosphorylcholine) (CTP-g-PMPC)* star copolymer with terminal Pg groups was synthesized by polymerizing MPC in the presence of hexavalent RAFT agent CTP-(CTA-COOH)_6_ as follows: A mixture of CTA-(CPADTB)_6_ (12.6 mg, 5.7 µmol) and AIBN (2.5 mg, 15.7 µmol) was dissolved in DMAc (1.5 mL) and then added to a solution of MPC (200.0 mg, 0.68 mmol) in methanol (5.3 mL). The solution was thoroughly bubbled with argon and polymerized in a sealed glass ampoule at 70 °C for 16 h. The solution was precipitated into acetone (100 mL) to give 132.6 mg of crude polymer as a pink powder. The polymer was dissolved in DMAc (1.3 mL) followed by the addition of 15.7 mg of ACVA-Pg; the mixture was then allowed to react at 80 °C for 3 h in a sealed glass ampoule. The polymer was isolated by precipitation into acetone (40 mL), with the solid content dissolved in PBS buffer (0.15 M, pH 7.4). The crude product was purified by membrane filtration using RC centrifugal filter units with MWCO 100 kDa in PBS (4×) and in H_2_O (2×). The purified star polymer (CTP-*g*-PMPC-Pg) was isolated from the aqueous solution by lyophilization to give 93.0 mg of a white powder. The *M*_n_, and *Ð* were 58.6 kg·mol^−1^ and 1.10, respectively. ^31^P NMR (162 MHz, D_2_O): *δ* 0.16 (s) ppm. The reaction scheme for the preparation of polymer (**2c**) is shown in Scheme 10.

**Scheme 11:**
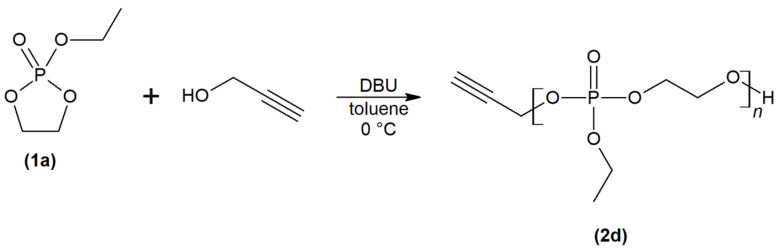
Reaction scheme for the synthesis of poly(ethyl ethylenephosphate) linear polymer with a terminal Pg group (**2d**).

*(**2d**) Poly(ethyl ethylenephosphate) (PEEP)* linear polymer with a terminal Pg group was synthesized according to a modified procedure described elsewhere [1]. Briefly, 2-ethoxy-2-oxo-1,3,2-dioxaphospholane (214.0 mg, 1.4 mmol) was introduced via cannula into a flame-dried glass vial under an argon atmosphere. Dry toluene (160 μL) and a solution of 2-propyn-1-ol in dry toluene (0.8 mg, 120 μL) were sequentially added and the reaction mixture subsequently cooled to 0 °C. The polymerization was initiated with the rapid addition of solution of DBU in dry toluene (10.7 mg, 120 μL). After 17 h at 0 °C, the polymerization was terminated by adding excess acetic acid (15 μL). The resulting polymer was obtained by precipitating the reaction mixture into diethyl ether (10 mL). After decanting the liquid layer, the precipitate was dried under vacuum to give 180 mg of PEEP-Pg polymer as a transparent viscous liquid. The *M*_n_ and *Ð* of PEEP-Pg were 3.7 kg·mol^−1^ and 1.03, respectively. ^31^P NMR (162 MHz, D_2_O): *δ* -0.06 (s) ppm. The reaction scheme for the preparation of polymer (**2d**) is shown in Scheme 11.

**Scheme 12:**
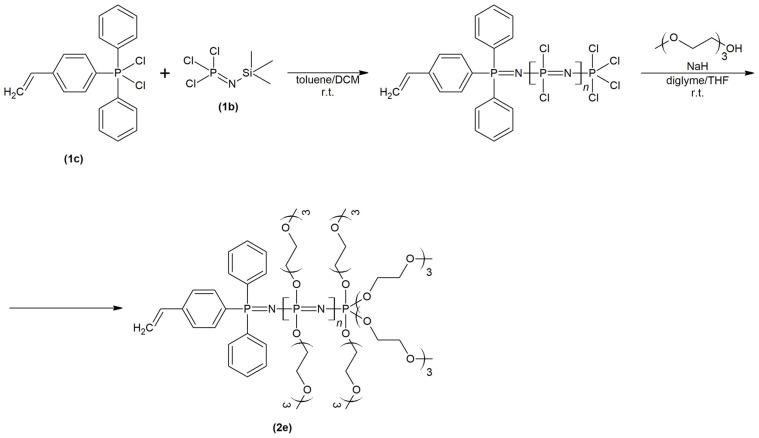
Reaction scheme for the synthesis of poly[bis(2-(2-(2-methoxyethoxy)ethoxy)ethoxy)]phosphazene linear polymer with a terminal vinyl group (**2e**).

*(**2e**) Poly[bis(2-(2-(2-methoxyethoxy)ethoxy)ethoxy)]phosphazene (PMEEEP)* linear polymer with a terminal vinyl (vi) group was synthesized by post-polymerization modification of a poly(dichlorophosphazene) precursor with 2-(2-(2-ethoxyethoxy)ethoxy)ethanol as follows: Firstly, a solution of 4-(dichlorodiphenylphosphino)styrene (0.1 mmol) in dry DCM (1.5 mL) was added dropwise to trichloro(trimethylsilyl)phosphoranimine (5.0 mmol) in dry toluene (20 mL) at 0 °C. The mixture was heated to r.t. and stirred for another 21 h. The solution was then filtered and concentrated. The prepared poly(dichlorophosphazene) precursor was re-dissolved in dry diglyme (6.27 mL) and stored in a freezer. Secondly, 2-(2-(2-methoxyethoxy)ethoxy)ethanol (2.5 mmol, 0.4 mL) was added portion-wise to a suspension of sodium hydride (2.5 mmol, 63.0 mg) in dry THF (4 mL) and the mixture then stirred at r.t. for 1 h. To this mixture, poly(dichlorophospahzene) (100 mg·mL^−1^ in diglyme, 0.5 mL) was added dropwise and stirred at r.t. for 17 h. The reaction was quenched by the addition of water (2 mL), with volatiles evaporated under reduced pressure. The crude polymer was purified by column chromatography using a Sephadex G-25 cartridge in water. The purified PMEEEP-Vi polymer was obtained from the aqueous solution by lyophilization to give 97 mg of orange oil. The *M*_n_ and *Ð* were 22.0 kg·mol^−1^ and 1.20, respectively. ^31^P NMR (162 MHz, D_2_O): *δ* -6.09 (s) ppm. The reaction scheme for the preparation of polymer (**2d**) is shown in Scheme 12.

## 4. Methods

### 4.1. UV–Vis Spectrophotometry

Spectrophotometric analysis of functionalized linear polymers was carried out in quartz glass cuvettes on the SPECORD PLUS UV–Vis spectrophotometer (Analytik Jena, Jena, Germany). The molar content of terminal DTB and TT groups in polymers was determined at 302 and 305 nm, respectively, in methanol using molar absorption coefficients of 12 100 and 10 300 L·mol^−1^·cm^−1^, respectively. The functionality (*f*) of the polymer (i.e., the average number of functional end groups per polymer chain) was calculated as the ratio of *M*_n_ of the polymer determined by GPC to *M*_n_ of the polymer determined by end group analysis using UV–Vis spectrophotometry.

### 4.2. Size-exclusion Chromatography

The number- and weight-averages of molecular weights (*M*_n_ and *M*_w_) and dispersities (*Ð*, *Ð = M*_w_*/M*_n_) for the polymer probes were determined by size-exclusion chromatography (SEC) on an HPLC system (Shimadzu, Kyoto, Japan) equipped with an internal UV–Vis diode array detector (SPD-M20A), an external differential refractometer (Optilab T-rEX, Santa Barbara, CA, USA), and a multiangle light scattering detector (DAWN HELEOS II, both Wyatt Technology, Santa Barbara, CA, USA). TSKgel SuperAW3000 and SuperAW4000 columns (Tosoh Bioscience, USA) connected in series were used to analyze samples in the mobile phase of 80 % methanol and 20 % sodium acetate buffer (0.3 m, pH 6.5) at a flow rate of 0.6 mL·min^−1^. The d*n*/d*c* values of 0.125, 0.104, and 0.120 mL·g^−1^ were used to calculate the molecular weights of the PMPC, PEEP, and PMEEEP polymers, respectively.

### 4.3. High-performance Liquid Chromatography

The purity of low-molecular-weight compounds was verified on a high-performance liquid chromatography (HPLC) system (Shimadzu, Kyoto, Japan) equipped with an internal UV–Vis diode array (SPD-M20A) and ELSD (LTII) detectors using the Chromolith HighResolution RP-18e reverse-phase column (Merck, Rahway, NJ, USA), with a linear gradient (0-100%) of a water–acetonitrile mixture containing 0.1% TFA at a flow rate of 2.5 mL·min^−1^.

### 4.4. Nuclear Magnetic Resonance

The structures of low-molecular-weight compounds were investigated by ^1^H and ^31^P nuclear magnetic resonance (NMR) spectroscopy in deuterated solvents on a Bruker DPX spectrometer (Bruker, Billerica, MA, USA) operating at 300.1 MHz. ^1^H NMR spectra were calibrated to the signal from the internal standard tetramethylsilane (*δ* = 0.00 MHz); ^31^P NMR were externaly referenced to the H_3_PO_4_ signal (*δ* = 0.00 MHz) shortly before the spectra were collected.

### 4.5. Magnetic Resonance Spectroscopy, Imaging and Relaxometry

MR characterization of the polymer probes was performed in aqueous solutions at a normalized phosphorus concentration of 100 mmol·L^−1^ using a 4.7 T scanner (Bruker BioSpin, Ettlingen, Germany) and a homemade dual ^1^H/^31^P radiofrequency (RF) surface coil as previously described [22].

^31^P MR spectroscopy measurements were performed using single-pulse sequences (flip angle FA = 90°, repetition time TR = 200 ms) with a varying number of averages corresponding to the acquisition time AT = 6 min to 1 h. Spectra were processed in Matlab software (Matlab v7.5.0.342, MathWorks, Natick, MA, USA) and quantified by comparing signal-to-noise ratio (SNR).

^31^P MR images were obtained using a chemical shift imaging (CSI) sequence (FA = 90°, TR = 500 ms, AT = 15 min to 3 h, field of view FOV = 36 mm^3^; resolution 2.25 × 2.25 × 5.8 mm^3^). ^31^P MRI SNR was calculated using SNR = 0.655 · *S* · *σ*^−1^, where *S* is signal intensity in the region of interest (ROI), *σ* is the standard deviation of background noise, and constant 0.655 reflects the Rician distribution of background noise in a magnitude MR image. The chemical shift of PTMPC relative to the reference PMPC was measured using a single-pulse sequence with the bandwidth covering the spectra of both polymers (16 181.2 Hz: 199.38 ppm).

^31^P relaxometry was performed using spectroscopy sequences; the ^31^P *T*_1_ relaxation time was measured using 10 spectroscopic single-pulse sequences with varying repetition times (TR = 200–4000 ms) and the ^31^P *T*_2_ relaxation time was measured using 10 spectroscopic Carr–Purcell–Meiboom–Gill (CPMG) sequences with varying echo times (TR = 5000 ms, TE = 20–1600 ms). Data were quantified by plotting amplitudes and fitting the appropriate curve (*S* ≈ *S*_0_ · (1 − e^−*t*/*T*1^) for *T*_1_; *S* ≈ *S*_0_ · e^−*t*/*T*2^ for *T*_2_), where *S* is signal intensity (*S*_0_ signal intensity at equilibrium) and *t* is time: TR for *T*_1_ and TE for *T*_2_, respectively.

## 5. Conclusions

In summary, we synthesized, and compared the MR properties of five distinct water-soluble biocompatible phosphopolymers (differing in composition, structure, and molecular weight) to evaluate their use as probes in ^31^P magnetic resonance imaging. Specifically, we used controlled polymerization techniques to prepare highly defined materials based on linear poly(2-methacryloyloxyethyl phosphorylcholine) (PMPC), poly(ethyl ethylenephosphate) (PEEP), and poly[bis(2-(2-(2-methoxyethoxy)ethoxy)ethoxy)]phosphazene (PMEEEP) as well as star-shaped copolymers composed of PMPC arms grafted onto poly(amidoamine) dendrimer (PAMAM-*g*-PMPC) or cyclotriphosphazene-derived cores (CTP-*g*-PMPC) with a molecular weight ranging from ~4–400 kg·mol^−1^. In phantom experiments, all polymer probes were detected at the magnetic fields close to those used in clinical practice. These novel probes provided satisfactory SNR values based on both ^31^P MR spectroscopy (209.8–12.1) and imaging (10.4–1.1). Linear PMPC and PMEEEP achieved the best results followed by star PAMAM-*g*-PMPC, CTP-*g*-PMPC, and linear PEEP. The ^31^P *T*_1_ and *T*_2_ relaxation times of the polymer probes were within a range of 1078–2368 ms and 30–171 ms, respectively, ensuring efficient detection of MR signals. The synthesized phosphopolymers offer great promise as biocompatible, structurally tunable, and long-circulating probes for in vivo ^31^P MRI. In addition, due to the presence of functional groups at the ends of the polymer chains, it is possible to introduce molecules with biological and/or contrasting functions (i.e., targeting units, fluorescent labels, drugs) into their structures, reflecting their therapeutic as well as diagnostic importance.

## Figures and Tables

**Table 1:** SEC and ^31^P MR characteristics of polymer probes (**2a–e**). The concentration of phosphorus in polymer probes used in all MR experiments was 100 mmol·L^−1^. All MR images were post-processed in the same way.

Polymer Probe	(2a)	(2b)	(2c)	(2d)	(2e)
Structure	IBN-PMPC-Pg	PAMAM-*g*-PMPC-Pg	CTP-*g*-PMPC-Pg	PEEP-Pg	PMEEEP-Vi
*M*_n_ [kg·mol^−1^]	18.8	398.8	58.6	3.7	22.0
*Ð*	1.06	1.10	1.10	1.03	1.20
SNR (^31^P MRSI)	3.52	1.39	1.54	1.11	10.4
SNR (^31^P MRS)	209.8	55.7	43.6	12.1	61.6
^31^P *T*_1_ [ms]	1078.2	1433.7	1454.1	2367.8	1139.4
^31^P *T*_2_ [ms]	58.3	78.3	170.6	113.4	30.3
^31^P MRSI	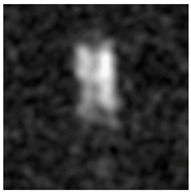	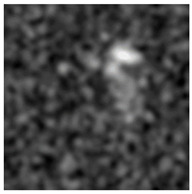	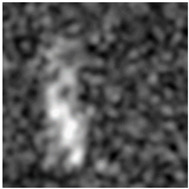	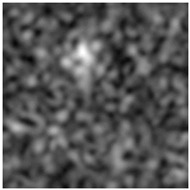	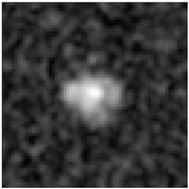

## Data Availability

Data available within the article or its Supplementary Materials.

## Supplementary Materials

The following supporting information can be downloaded at: https://www.mdpi.com/article/10.3390/molecules28052334/s1, Figure S1: 31P NMR spectrum of polymer (2a) in D2O; Figure S2: 31P NMR spectrum of polymer (2b) in D2O; Figure S3: 31P NMR spectrum of polymer (2c) in D2O; Figure S4: 31P NMR spectrum of polymer (2d) in D2O; Figure S5: 31P NMR spectrum of polymer (2e) in D2O; Figure S6: SEC profiles of 31P-MR probes; Figure S7: Comparison of the sensitivities of 31P MR measurements of PMPC polymer probe using 31P CSI and 31P RARE sequences; Figure S8: Comparison of the sensitivities of ^31^P MRI/MRSI measurements of PMPC polymer probe using ^31^P RARE and ^31^P CSI sequences.

## Sample Availability

Samples of the compounds are not available from the authors.
