# Supplementary material for: Phosphorus-Containing Polymers as Sensitive Biocompatible Probes for 31P Magnetic Resonance
Source: Molecules. 2023 Mar 2;28(5):2334. doi: 10.3390/molecules28052334 (PMC10005191; doi:10.3390/molecules28052334)
Supplement: Supplementary file 1 [file molecules-28-02334-s001.zip › molecules-2103570-supplementary.pdf]

molecules

Supplementary Materials

Phosphorus-Containing Polymers as Sensitive Biocompatible Probes for ^{31}P Magnetic Resonance

Lucie Kracíková [1,2], Ladislav Androvič [1], Iveta Potočková [1], Natalia Ziółkowska [3], Martin Vít [3,4], David Červený [3,4], Daniel Jirák [3,4,5] and Richard Laga [1,*]

1 Institute of Macromolecular Chemistry, Czech Academy of Sciences, Heyrovského nám. 2, 162 06 Prague, Czech Republic
2 Department of Polymers, Faculty of Chemical Technology, University of Chemistry and Technology, Technicka 5, 166 28 Prague, Czech Republic
3 Institute for Clinical and Experimental Medicine, Vídeňská 1958/9, 140 21 Prague, Czech Republic
4 Institute of Biophysics and Informatics, First Faculty of Medicine, Charles University, 121 08 Prague, Czech Republic
5 Faculty of Health Studies, Technical University of Liberec, Studentská 1402/2, 461 17 Liberec, Czech Republic
* Correspondence: laga@imc.cas.cz; Fax: +420-296-809-410

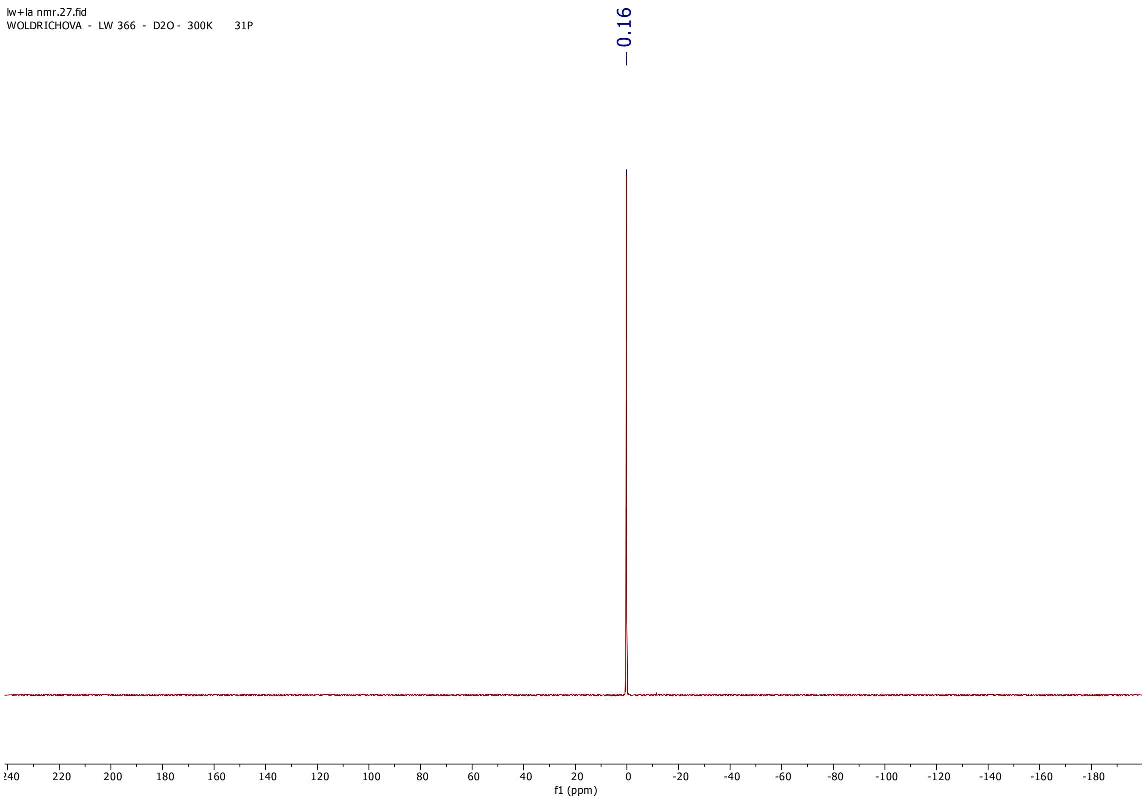

Figure S1. ^{31}P NMR spectrum of polymer (**2a**) in D_2O.

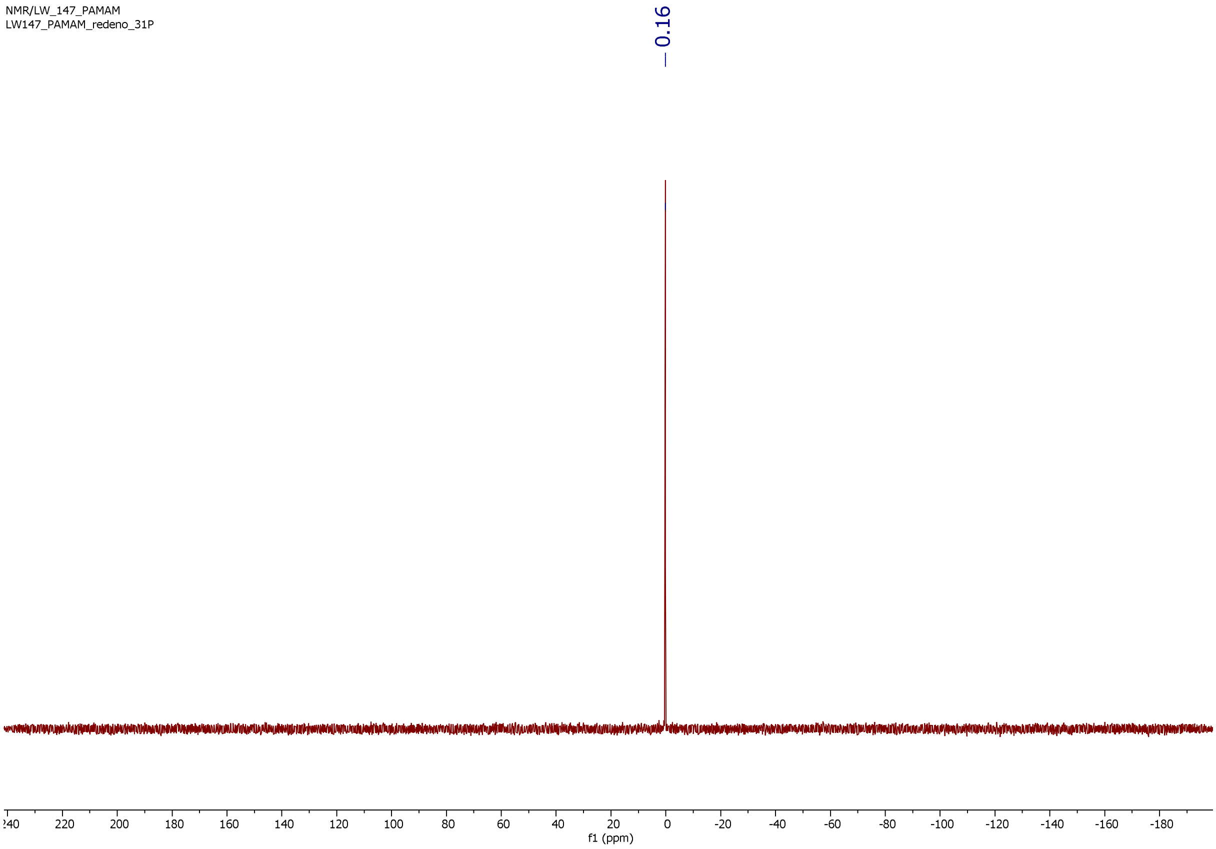

Figure S2. ^{31}P NMR spectrum of polymer (**2b**) in D_2O.

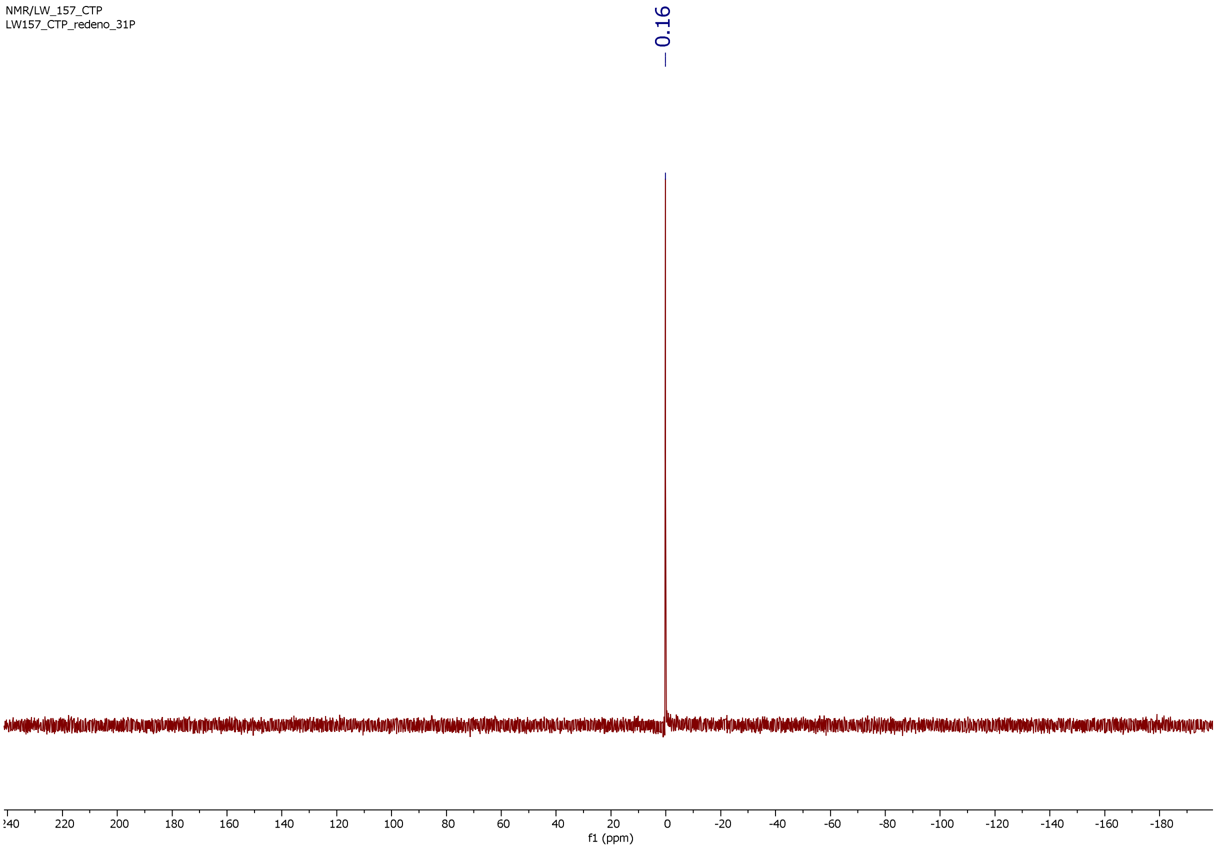

Figure S3. ^{31}P NMR spectrum of polymer (**2c**) in D_2O.

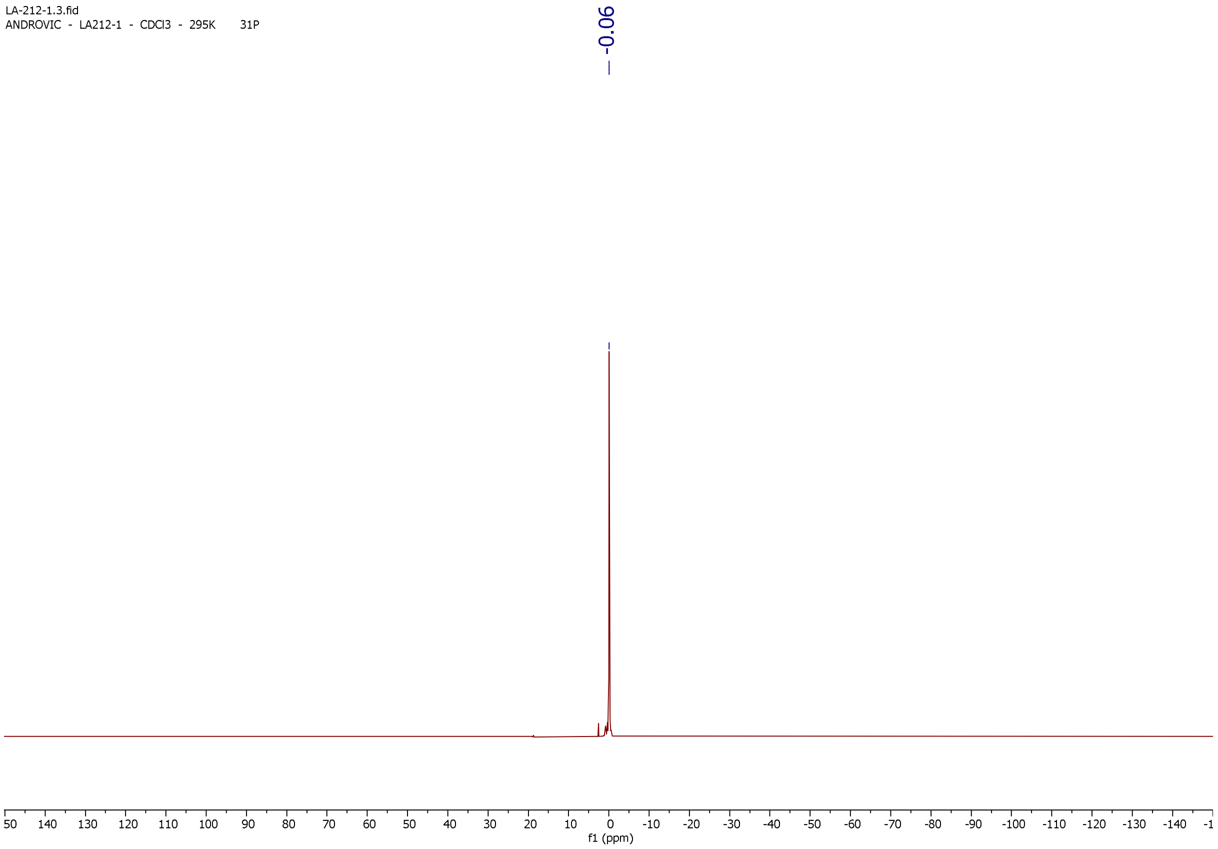

Figure S4. ^{31}P NMR spectrum of polymer (**2d**) in D_2O.

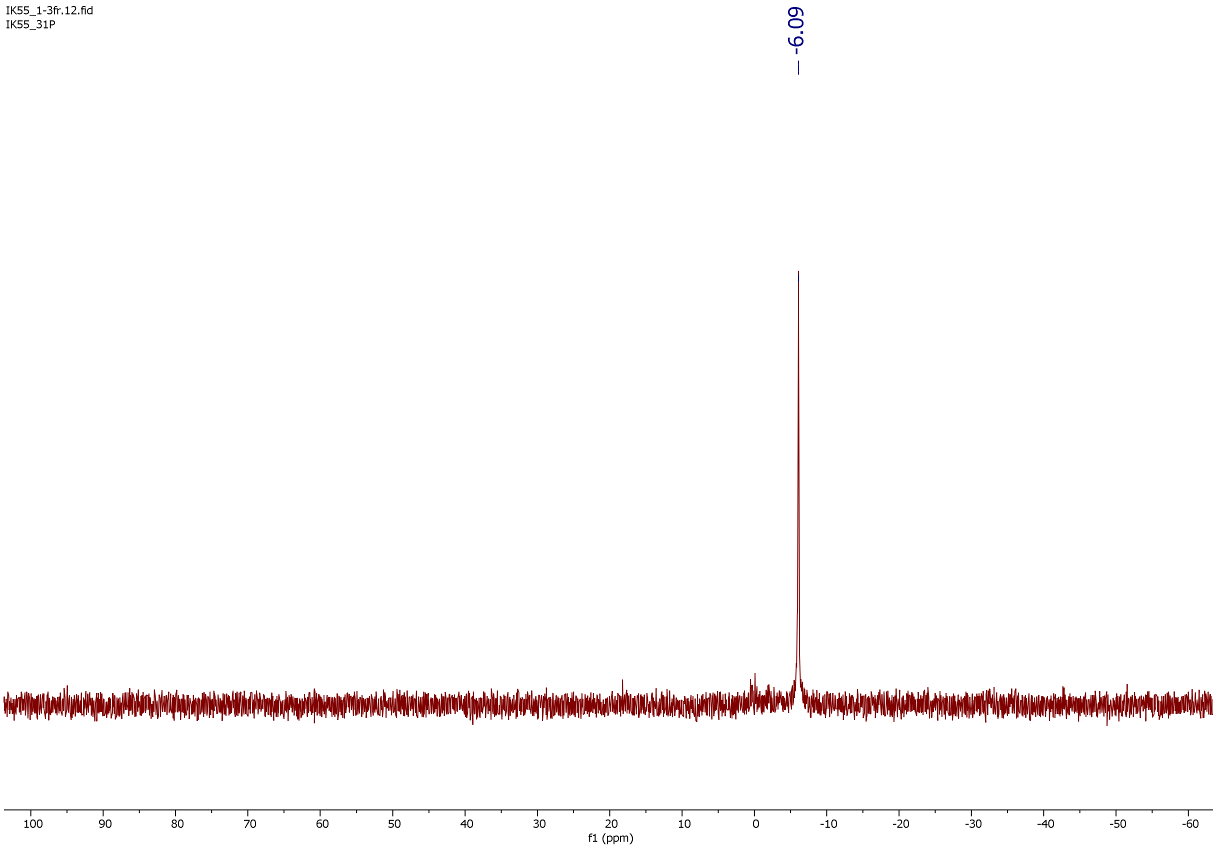

Figure S5. ^{31}P NMR spectrum of polymer (**2e**) in D_2O.

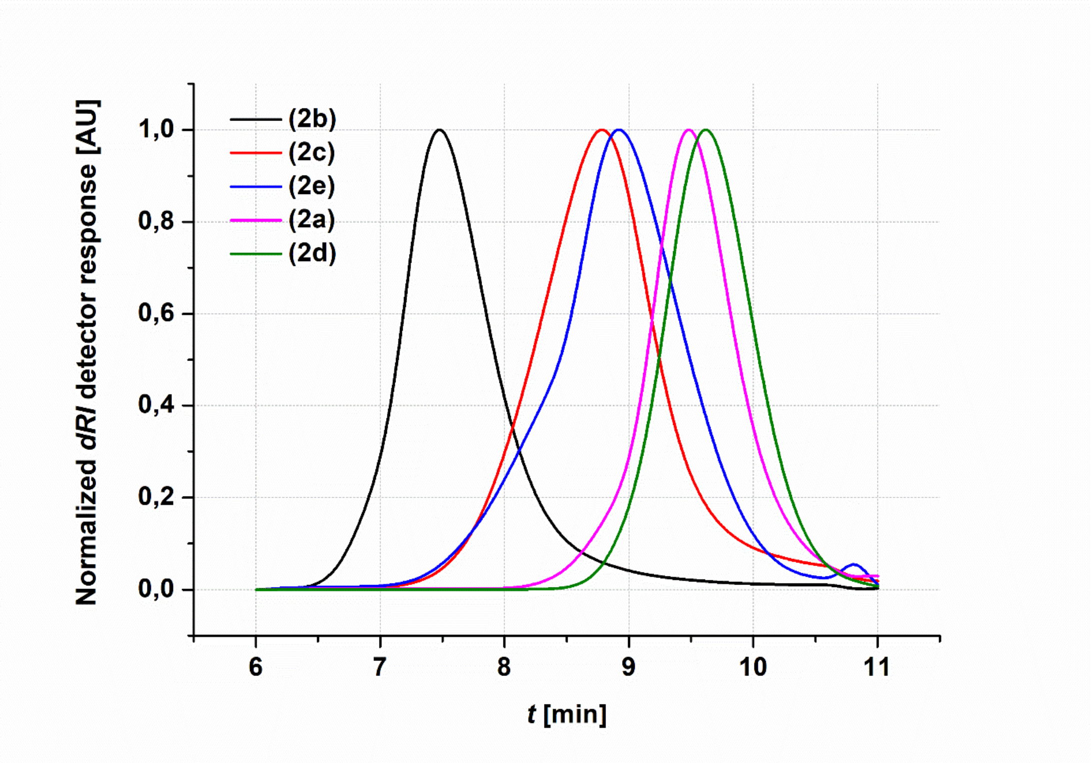

Figure S6. SEC profiles of ^{31}P-MR probes: (**2a**) IBN-PMPC-Pg, (**2b**) PAMAM-g-PMPC-Pg, (**2c**) CTP-g-PMPC-Pg, (**2d**) PEEP-Pg and (**2e**) PMEEEP-Vi.

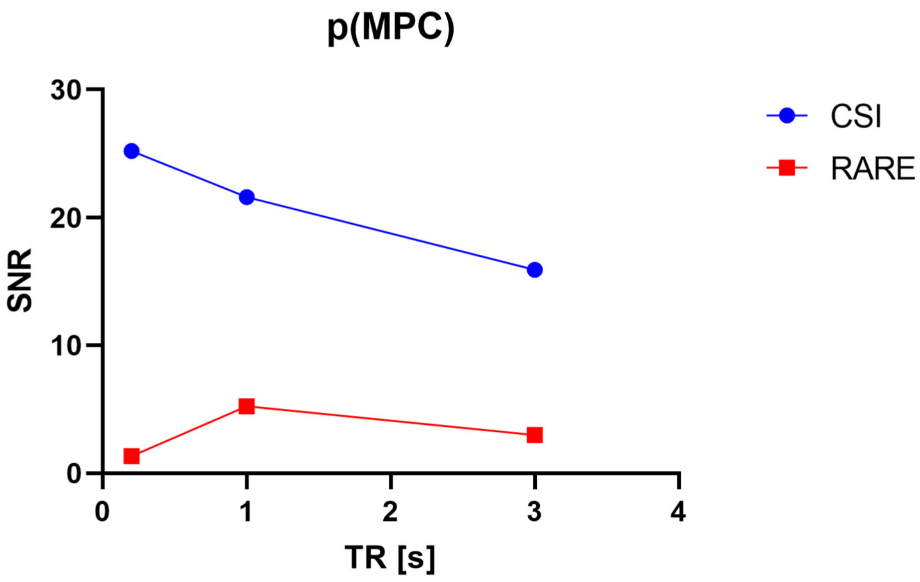

Figure S7. Comparison of the sensitivities of ^{31}P MR measurements of PMPC polymer probe using ^{31}P CSI (blue line) and ^{31}P RARE sequences (red line), at constant measurement time with varying TR.

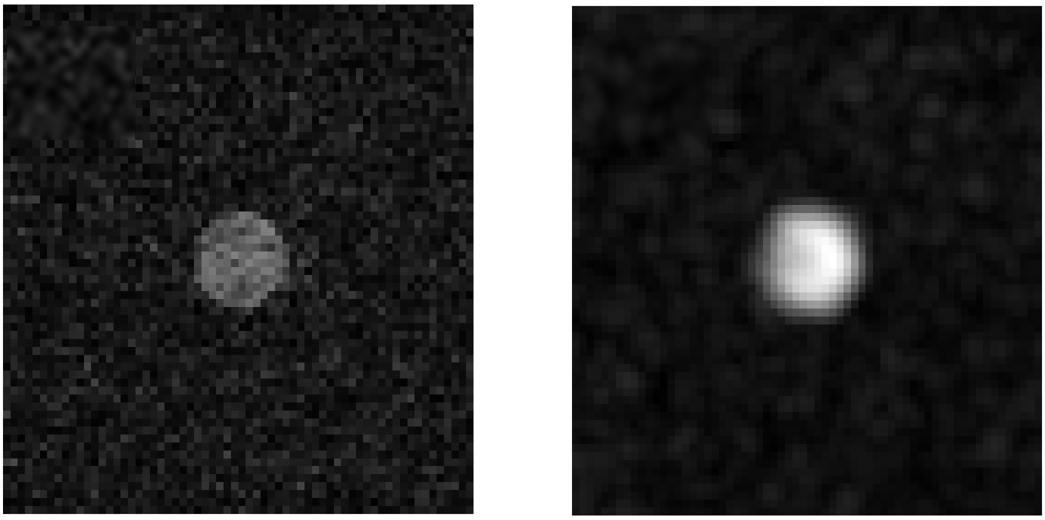

Figure S8. Comparison of the sensitivities of ^{31}P MRI/MRSI measurements of PMPC polymer probe using ^{31}P RARE (A) and ^{31}P CSI (B) sequences. Images were acquired with the same repetition time (TR) and geometry/resolution (TR = 1s, FOV = 4 x 4 cm, slice thickness = 1 cm); No post-process filtration was performed.
